# Pregnancy Outcomes and Associated Complications in Patients Undergoing Hemodialysis and Their Neonates: A Nationwide Study in South Korea (2014–2022)

**DOI:** 10.3390/jcm15124621

**Published:** 2026-06-14

**Authors:** Jee Young Lee, Sang Hyun Park, Hye Won Park, Kyung Won Kim, Tae-Eun Kim

**Affiliations:** 1 Devision of Nephrology, Department of Internal Medicine, Konkuk University School of Medicine, Konkuk University Medical Center, Seoul 05030, Republic of Korea; 2 Department of Clinical Pharmacology, Konkuk University Medical Center, Seoul 05030, Republic of Korea; 3 Department of Pediatrics, Konkuk University Medical Center, Konkuk University School of Medicine, Seoul 05030, Republic of Korea; 20110673@kuh.ac.kr; 4 Devision of Nephrology, Department of Internal Medicine, Konkuk University Medical Center, Seoul 05030, Republic of Korea

**Keywords:** end-stage kidney disease, pregnancy, hemodialysis, pregnancy outcomes, congenital anomalies

## Abstract

**Introduction**: Pregnancy in women with end-stage kidney disease (ESKD) remains rare and high-risk, despite advancements in dialysis and supportive care. Using a nationwide database in South Korea, this study examined the maternal and neonatal outcomes among women undergoing maintenance hemodialysis, with a particular focus on dialysis modality and treatment patterns. **Methods**: This population-based retrospective cohort study utilized data from the Korean National Health Insurance Service database. The study included all live births between 1 January 2014 and 31 December 2022, linked to mothers who underwent hemodialysis at least twice per week during pregnancy. **Results**: Between 2014 and 2022, in the Republic of Korea, 31 live births were recorded among 29 women undergoing hemodialysis. The mean maternal age at delivery was 36.1 ± 4.94 years, and most patients had significant comorbidities, including hypertension (79.3%), and diabetes mellitus (48.3%). Cesarean section was the predominant mode of delivery (75.9%). Pregnancy-related complications included preterm delivery (48.4%), preeclampsia (16.1%), and gestational diabetes (16.1%). A total of 16.1% of the neonates had atrial septal defects. During the peripartum period, 93.1% of deliveries occurred at tertiary care centers, and trimester-wise escalation in dialysis frequency was observed. **Conclusions**: This study provided real-world data on pregnancy-related outcomes among women with ESKD undergoing maintenance dialysis in Korea. Given the rarity of this clinical condition, our findings may serve as a valuable reference for the management of pregnant women with ESKD.

## 1. Introduction

Advances in dialysis techniques and pharmacological therapies have significantly improved the survival and quality of life in patients with end-stage kidney disease (ESKD) [1]. Despite these advancements, patients with ESKD still face numerous medical and psychosocial challenges that differ markedly from those encountered in the general population. Among these challenges, pregnancy and childbirth represent particularly complex and high-risk situations due to altered physiological states, immunosuppressive therapies, and complications related to renal failure. In addition, limited fertility and an increased risk of adverse maternal and fetal outcomes necessitate individualized specialized management approaches throughout pregnancy.

In patients with ESKD, pregnancy is rare due to multiple hormonal disturbances. Chronic kidney dysfunction disrupts the hypothalamic–pituitary–gonadal axis, leading to impaired secretion of gonadotropin-releasing hormone (GnRH), luteinizing hormone (LH), and follicle-stimulating hormone (FSH) [2]. This results in anovulation and menstrual cycle irregularities. Additionally, low estradiol and elevated levels of prolactin—commonly observed in ESKD due to reduced renal clearance—further suppress gonadotropin secretion and impair reproductive function [3]. Chronic hypoestrogenism also causes endometrial atrophy, which makes implantation difficult [4].

These multifactorial barriers highlight the complex reproductive challenges faced by women with ESKD, despite medical advances that support successful pregnancy. Beyond hormonal factors, impaired renal clearance of toxins, chronic inflammation, and vascular dysfunction are also commonly observed in patients with ESKD. These factors increase the likelihood of maternal complications such as hypertension, preeclampsia, anemia, and maternal morbidity and mortality, as well as adverse fetal outcomes, including preterm birth, low birth weight, and intrauterine growth restriction [5,6]. Specialized care is essential to optimize outcomes for both mothers and babies.

Although pregnancy in patients with ESKD was once considered almost impossible, improvements in renal replacement therapies and transplantation have expanded these possibilities. Kidney transplantation (KT) is considered the most favorable treatment option for women with ESKD planning pregnancy, as it offers improved fertility and is associated with significantly lower rates of preterm birth and maternal complications compared to dialysis [7]. However, KT has its own limitations, including the scarcity of donor organs, the necessity of postponing pregnancy for at least 1 year post-transplantation to minimize risks, and the uncertain impact of pregnancy on graft survival and function [8]. Peritoneal dialysis (PD) offers an alternative renal replacement modality during pregnancy; however, studies indicate that infants born to mothers on PD may have suboptimal growth parameters, such as being small for their gestational age, compared to those on hemodialysis [9]. As a result, despite KT being the most favorable option in theory, hemodialysis remains the primary and most feasible treatment modality for pregnant women with ESKD in real-world clinical practice.

Although significant progress has been made in understanding pregnancy in women with ESKD, comprehensive data on pregnancy outcomes remain scarce, particularly in certain populations and geographic regions. A recent systematic review of chronic dialysis patients published between 2010 and 2020—including 2364 women and 2754 pregnancies from 11 countries across six continents—reported that although risks remain high, the number of pregnancies and live-birth outcomes have increased, with live birth rates substantially higher than historically reported [10]. Moreover, recent nationwide registry- and survey-based studies in individual countries (e.g., in France and Japan) further confirm this upward trend, demonstrating that pregnancy and live birth are increasingly feasible in women on long-term hemodialysis under careful management. Despite this global progress, in South Korea, there remains a distinct lack of large-scale, population-based data on pregnancy outcomes in ESKD patients undergoing hemodialysis—most reports are limited to small case series or single-center experiences. Therefore, recognizing this critical gap, we designed the present study to better define the current status and to lay the groundwork for enhanced care, with the hope that our findings will ultimately contribute to improving treatment and support for hemodialysis patients in Korea who may wish to pursue pregnancy and childbirth.

## 2. Methods

### 2.1. Data Sources and Study Population

**Figure 1:**
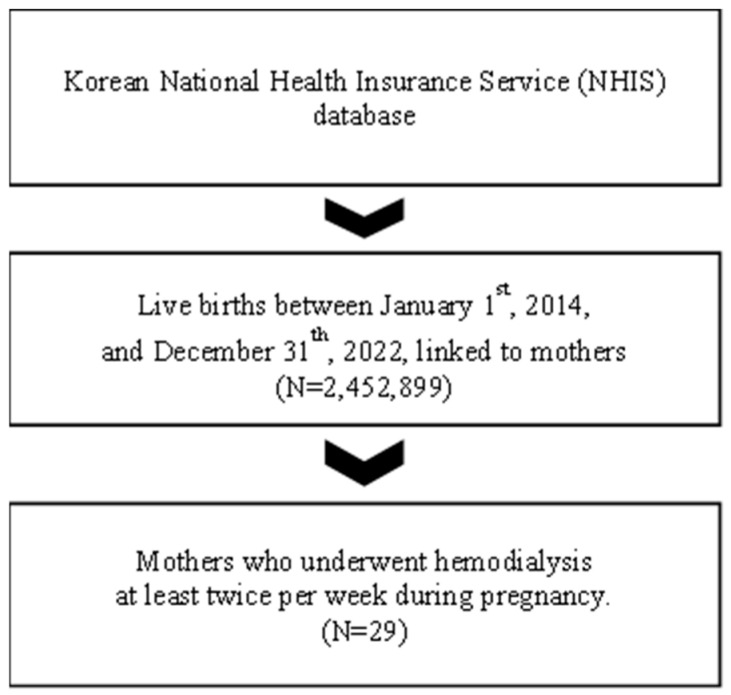
Flow chart of patient inclusion.

This study utilized population-based data from the Korean National Health Insurance Service (NHIS) database, which covers more than 97% of the South Korean population. This large-scale database includes detailed records of demographic characteristics, medical history, treatment, and prescriptions. Diagnoses were recorded using the International Classification of Diseases, 10th Revision (ICD-10). All data were de-identified by the NHIS to ensure patient confidentiality. The study population consisted of a mother–offspring cohort derived from the NHIS database, including all newborns in South Korea between 1 January 2014, and 31 December 2022, linked to their mothers. Mothers who underwent hemodialysis at least twice per week during pregnancy and their offspring were included in this study (Figure 1). This study protocol was approved by the NHIS of Korea and the Institutional Review Board of Konkuk University Medical Center (IRB No. KUMC 2025-07-056). The requirement for informed consent was waived by the IRB because this study analyzed de-identified, secondary administrative data. All procedures were performed in accordance with the relevant guidelines and regulations, including Article 32 of the Declaration of Helsinki.

### 2.2. Definitions of Variables

ESKD mothers undergoing maintenance hemodialysis were identified using a special exemption code (V001), indicating eligibility for hemodialysis coverage by the special cases for calculation of co-payment for health insurance. Hemodialysis sessions, details of vascular access, and delivery methods were identified using the NHIS procedure codes for the special cases to calculate the co-payment for health insurance. Maternal chronic diseases and pregnancy-related complications were defined using ICD-10 codes, whereas KT was identified using procedure codes. We defined the index date as the date of delivery. For maternal chronic diseases, the presence of conditions was ascertained using a look-back period extending from 1 year prior to conception through the index date. Pregnancy-related complications were identified using codes recorded specifically during the gestational period. In all cases, definitions required the presence of at least one corresponding primary or secondary diagnosis code. Major congenital anomalies in the offspring were determined using one or more ICD-10 codes recorded during the first year of life (from the index date to 1 year postpartum). Detailed code numbers are provided in Supplementary Material (Table S1).

### 2.3. Statistical Analysis

Descriptive statistics were used to summarize all the variables of interest. Continuous variables were reported as means and standard deviations (SD), while categorical variables were presented as frequencies and percentages. As the NHIS database does not include pregnancy onset dates, the last menstrual period (LMP) was estimated using a validated algorithm [11]. Weekly dialysis sessions were calculated for three distinct periods (preconception, gestation, and postpartum) by dividing the total number of hemodialysis sessions by the number of days in each period and multiplying by 7. The preconception period was defined as 365 days before the estimated LMP, the gestation period as the interval from the estimated LMP to the delivery date, and the postpartum period as 365 days after delivery. For patients initiating hemodialysis during pregnancy, the dose was calculated by dividing the number of hemodialysis sessions during pregnancy by the number of days between the first hemodialysis session and the delivery date, and then multiplying by 7. For the analysis of medical institution utilization during the preconception, gestation, and postpartum periods, the most frequently visited healthcare institution during each period was recorded for each patient and categorized as primary, secondary, or tertiary, based on the Korean healthcare system. All statistical analyses were performed using SAS version 9.4 (SAS Institute Inc., Cary, NC, USA).

## 3. Results

Between 2014 and 2022, 31 childbirths, resulting in 31 live births, were recorded among 29 women undergoing hemodialysis in the Republic of Korea. Table 1 summarizes the patients’ baseline characteristics. The mean maternal age at delivery was 36.1 ± 4.94 years. The dialysis duration before delivery was less than 1 year in eight patients (27.6%), 1–2 years in four patients (13.8%), 2–3 years in three patients (10.3%), 3–4 years in four patients (13.8%), and more than 4 years in 10 patients (34.5%). Regarding vascular access, 18 patients (62.1%) used either a hemocatheter or a permanent catheter, while 14 patients (48.1%) had an arteriovenous (AV) fistula. None of the patients underwent AV grafts. The overlapping percentages suggest that some patients may have transitioned from a catheter to an AV fistula, or had both types of access during the course of their pregnancy.

In terms of comorbidities, 23 patients (79.3%) had hypertension, 14 (48.3%) had diabetes mellitus, 26 (89.7%) had dyslipidemia, three (10.3%) had coronary artery disease, seven (24.1%) had heart failure, two (6.9%) had chronic airway disease such as chronic obstructive pulmonary disease or asthma, six (20.7%) had liver disease such as hepatitis C virus or hepatitis B virus or liver cirrhosis, and two (6.9%) had systemic lupus erythematosus. None of the patients had autosomal dominant polycystic kidney disease (ADPKD), human immunodeficiency virus (HIV) infection, or acquired immunodeficiency syndrome (AIDS). A history of kidney transplantation was identified in two patients (6.9%).

Table 2 summarizes the clinical characteristics associated with pregnancy in 29 patients with ESKD. Among the 29 pregnancies, two involved multiple gestations, indicating a 6.9% incidence of twin pregnancies. Cesarean section was the predominant mode of delivery (75.9%, *n* = 22), while spontaneous vaginal delivery occurred in 24.1% (*n* = 7) of the cases, suggesting a strong preference for or indication for surgical delivery, likely due to high-risk maternal or fetal conditions related to dialysis. An analysis of conception timing relative to dialysis initiation showed that most pregnancies (75.9%, *n* = 22) occurred after dialysis initiation, whereas only 24.1% (*n* = 7) occurred before dialysis initiation.

During the 10-month pregnancy period, patients had a mean of 121.8 ± 58.8 outpatient visits, and an average of 2.93 ± 1.86 times of hospitalizations, with dialysis sessions included in the outpatient visits. Delivery was primarily performed in tertiary care hospitals (93.1%), followed by secondary care hospitals (6.9%), highlighting the importance of high-level perinatal and nephrological expertise in managing these cases. Weekly dialysis sessions significantly increased during pregnancy, with the mean number of sessions rising from 2.85 ± 0.34 pre-pregnancy to 3.93 ± 0.91 during pregnancy, then returning to 2.83 ± 0.43 postpartum. This pattern reflects the clinical aim of optimizing volume status and solute clearance during gestation. A trimester-specific analysis showed a progressive increase in dialysis frequency: 2.88 ± 0.40 sessions per week in the first trimester, 3.92 ± 1.94 in the second trimester, and 4.51 ± 1.07 in the third trimester. Regarding long-term renal replacement strategies, kidney transplantation was performed in 27.6% in the postpartum period. PD was initiated in 10.3% of patients after delivery. All patients continued hemodialysis after childbirth, except for those who transitioned to peritoneal dialysis or underwent kidney transplantation.

Table 3 illustrates the distribution of hemodialysis centers used during the peri-pregnancy period. Primary care centers provided the majority of dialysis treatments 1 year before pregnancy (57.1%) and 1 year after pregnancy (50.0%), indicating that most patients generally received dialysis in lower-level care settings during stable periods. Although the proportion of patients receiving dialysis at primary care centers decreased during pregnancy (44.8%), these facilities remained the most frequently utilized, suggesting a continued reliance on local care despite increased clinical complexity. In contrast, the utilization of tertiary care centers increased markedly during pregnancy, rising from 14.3% pre-pregnancy to 34.5% during pregnancy.

Table 4 presents pregnancy-related maternal complications observed in the study population. Among the 31 pregnancies, preeclampsia and gestational diabetes were each reported in 17.2% of cases (*n* = 5). Other antepartum complications included premature rupture of membranes (PROM) (13.8%), genitourinary infection (13.8%), and abnormal uterine bleeding (10.3%). Regarding delivery-related complications, preterm delivery was the most prevalent, occurring in 51.7% of the pregnancies (*n* = 15), underscoring the high burden of prematurity among patients undergoing dialysis. Fetal malpresentation was also relatively common (20.7%, *n* = 6), and false labor was documented in 13.8% (*n* = 4) of cases. Table 5 summarizes the incidence of congenital anomalies in neonates born to mothers undergoing hemodialysis. Of the 31 live births, five (16.13%) had congenital malformations, all of which were classified as cardiac defects, specifically atrial septal defects (ASDs). No anomalies have been reported in other organ systems, including the nervous and respiratory systems, gastrointestinal and genitourinary tracts, limbs, or craniofacial region.

## 4. Discussion

This study provides a descriptive analysis of clinical characteristics, healthcare utilization patterns, and maternal complications, as well as congenital anomalies in live-born infants, among pregnant women with ESKD undergoing maintenance hemodialysis in Korea. Since the first successful pregnancy in a patient on dialysis was reported in 1971, the number of successful pregnancies in this population has steadily increased worldwide, largely owing to advancements in dialysis technology and specialized care. Reflecting this global trend, reports of successful deliveries have been increasingly documented in the Korean clinical setting. Therefore, we analyzed recent cases of successful pregnancies among Korean women undergoing maintenance hemodialysis to better understand this unique and growing patient population.

The mean maternal age at delivery was 36.1 years, which is higher than that of the general obstetric population, likely reflecting delayed conception and the need for clinical stabilization before pregnancy [12]. Comorbidities were common in our cohort, with hypertension (79.3%), diabetes mellitus (48.3%), and dyslipidemia (89.7%) being the most prevalent conditions. Among the 29 patients with available dialysis vintage data, 27.6% had been on dialysis for less than 1 year before delivery, 13.8% for 1–2 years, 10.3% for 2–3 years, 13.8% for 3–4 years, and 34.5% for more than 4 years. These findings suggest that while pregnancy can occur relatively early after dialysis initiation, a substantial proportion of patients conceive after prolonged dialysis duration. Previous studies have reported dialysis vintages ranging from 1 to 12 years, with most pregnancies occurring in women with 1–3 years of dialysis history [6,13,14,15,16,17,18,19,20,21,22]. Overall, these data suggest that conception is possible across a wide range of dialysis durations, although it often occurs later than in the general population, likely due to both medical and physiological barriers.

In our study, two involved multiple gestations, resulting in two sets of twins, suggesting that even twin pregnancies are feasible despite the physiological stress associated with dialysis. An analysis of conception timing relative to dialysis initiation showed that most pregnancies (75.9%, *n* = 22) occurred after dialysis initiation, whereas only 24.1% (*n* = 7) occurred before dialysis initiation. This indicates that conception is more common among women already undergoing dialysis than among those who conceived before dialysis initiation. This result is consistent with the findings of a French study by Baouche et al., which reported that 83.9% of conceptions occurred in women receiving chronic dialysis, whereas 16.1% occurred before the initiation of dialysis [14]. However, this finding contrasts with the study by Jesudason et al., which analyzed data from the Australian and New Zealand Dialysis and Transplantation Registry (ANZDATA) [21]. They reported a higher live birth rate in women with ESKD who conceived before starting dialysis than in those who conceived after dialysis initiation. The authors suggested that preserved residual renal function might be a key factor contributing to improved pregnancy outcomes in women who conceived before dialysis. Several factors may explain the discrepancies between these two studies. First, Jesudason et al.’s study included more than twice the number of patients, which may have contributed to greater statistical power. Second, their cohort included patients undergoing both hemodialysis and PD, whereas our study was limited to patients undergoing hemodialysis-only. Third, differences in ethnicity and geographic location between the two populations may have influenced the maternal and fetal outcomes, given that genetic, cultural, and healthcare system factors can all play a role in pregnancy prognosis. Lastly, advancements in weekly dialysis sessions, obstetric care, and specialized management in recent years may have improved the outcomes in our cohort compared to the historical data used in the ANZDATA analysis.

The average number of hospitalizations during pregnancy (2.93 ± 1.86) highlights the intensive monitoring required for this high-risk population. Most patients (93.1%) delivered at tertiary care hospitals during the perinatal period. In contrast, only a small proportion (6.9%) delivered at secondary care hospitals. Consistently, there was a notable shift in dialysis care settings during pregnancy: the use of tertiary care centers increased from 14.3% pre-pregnancy to 34.5% during pregnancy, whereas the use of primary and secondary care decreased during pregnancy compared to the pre-pregnancy period. The increase in the use of superior medical facilities aligns with the report by the French study by Baouche et al., which found that in-center dialysis increased and home hemodialysis decreased after pregnancy compared to the pre-pregnancy period [14]. This highlights the clinical complexity and need for integrated nephrology–obstetric care, though interpretation is limited by the dataset capturing visit frequency rather than care intensity or severity. A small proportion of patients underwent KT before or after delivery, and some transitioned to KT (27.6%) or PD (10.3%). This suggests that the postpartum period serves as a critical window for reevaluating long-term renal replacement strategies. Pregnancy may act as a turning point in the trajectory of renal care, necessitating proactive planning for transplant evaluation and adjustment of dialysis modality.

The number of dialysis sessions increased during pregnancy (3.93 ± 0.91 per week), compared to the pre-pregnancy (2.85 ± 0.34) and postpartum (2.83 ± 0.43) periods. This adjustment aligns with the international recommendations to intensify dialysis during pregnancy to improve maternal volume status, reduce uremic toxins, and optimize fetal outcomes [23,24,25,26,27]. In our report, weekly dialysis sessions increased progressively across trimesters: 2.88 ± 0.40 sessions per week in the first trimester, 3.92 ± 1.94 in the second trimester, and 4.51 ± 1.07 in the third trimester. This trimester-specific increase reflects efforts to match weekly dialysis sessions with the growing physiological demands of pregnancy and to minimize potential maternal-fetal complications [6,14,15,16,17,19,20,28]. A comparable pattern of increasing dialysis with advancing gestational age has been reported in other studies. Hirano et al., in an analysis of pregnancy and delivery outcomes of patients with ESKD undergoing maintenance dialysis in Japan, observed that the average dialysis frequency increased from 4.2 sessions per week in the first trimester to 5.1 in the second and 5.4 in the third among the 20 pregnant women receiving maintenance hemodialysis. Similarly, a study by Baouche et al., which analyzed 240 pregnant patients with ESKD undergoing maintenance dialysis in France, found that the proportion receiving daily dialysis increased to 26%, 59.3%, and 55% in the first, second, and third trimesters, respectively [14]. Despite the trimester-wise escalation observed in our cohort, the overall dialysis frequency remained lower than that reported in the Japanese and French studies. This discrepancy is likely attributable to regional differences in clinical practice and institutional protocols regarding dialysis intensification during pregnancy, rather than differences in patient severity. 

In our study, the cesarean section rate was 77.4%, which may reflect both obstetric indications and precautionary decisions made in response to maternal comorbidities or fetal compromise. Previous studies have reported cesarean section rates ranging from 9% to 100% [6,15,16,17,18,19,20,22,28,29,30]. In our study, preeclampsia and gestational diabetes were each observed in 16.13% of cases, whereas infections and PROM affected 12.9% of pregnancies. The incidence of maternal complications in our study was higher than that in general pregnancies, probably due to higher maternal age and comorbidities [31]. These findings are consistent with existing literature, which indicates that pregnant women undergoing dialysis are at a significantly higher risk of hypertensive disorders, metabolic complications, and genitourinary infections. These risks are attributed to chronic inflammation, impaired immunity, and disruption of fluid and electrolyte homeostasis. Interestingly, the incidence of preeclampsia in our study was lower than that reported in some international studies [29,30], although it was higher than that reported in most other studies [14,17,19,20,21,28]. The incidence of PROM in our cohort was higher than that reported in most other countries [14,15,17,19,30]. This discrepancy could be attributed to several factors. First, unlike many other studies that included all pregnancies, our study was limited to women who delivered, potentially selecting more severe or advanced cases. Additionally, our cohort had a relatively higher maternal age, different dialysis protocols, and a smaller sample size, all of which may have influenced the observed rates of preeclampsia and PROM.

Among the neonates, the incidence of congenital anomalies was 16.1%, all of which were classified as ASDs. The observed clustering of ASD should be interpreted with caution. ASD is common among preterm infants. Given the high prevalence of preterm delivery in our cohort, this finding may partly reflect prematurity and the more intensive echocardiographic screening of high-risk neonates rather than a specific effect of maternal hemodialysis, and the small number of events precludes any causal inference. We therefore regard this observation as hypothesis-generating, warranting verification in larger, ideally pooled international cohorts. The strengths of this study include its use of a comprehensive national database, enabling robust identification of a rare clinical population and longitudinal assessment of maternal and neonatal outcomes. However, this study had a few limitations. First, this study was restricted to live births. Consequently, pregnancies resulting in stillbirth or spontaneous abortion were not included, which may have introduced a selection bias toward women with relatively favorable clinical courses and may have led to an underestimation of the overall adverse pregnancy outcomes in pregnant women with ESKD undergoing maintenance dialysis. Second, due to the inherent limitations of claims-based data, our dataset lacks clinical data such as residual renal function, laboratory values, and medication details were unavailable. Residual kidney function (e.g., residual urine output or residual renal clearance) is an important determinant of dialysis adequacy and pregnancy outcome, but it is recorded only in dialysis-unit clinical charts rather than in reimbursement data; this information was therefore unavailable for both patients who initiated hemodialysis during pregnancy and those already established on maintenance hemodialysis. Furthermore, the identification of diagnoses and clinical events relied solely on administrative codes, which may be subject to misclassification. Third, gestational age and timing of conception were estimated using established claims-based algorithms rather than exact clinical records, as the database does not capture gestational age in weeks or the last menstrual period directly. These estimates may therefore be subject to misclassification, and the exact gestational age at hemodialysis initiation among women who conceived before starting dialysis could not be precisely determined and was not reported. Fourth, the small sample size and the descriptive nature of the study without a comparator group limit the generalizability of the findings to a broader population. Fifth, the ascertainment of maternal baseline comorbidities, including kidney transplantation, was based on diagnostic codes recorded during the period from one year before conception through delivery. Although the conditions examined are chronic diseases for which pre-existing diagnosis is considerably more likely than de novo onset during gestation, it cannot be entirely excluded that some diagnoses were first established during the pregnancy period itself, which may have introduced a degree of misclassification in the characterization of baseline comorbidity.

Despite these limitations, this study provides real-world data on pregnancy-related outcomes in women with ESKD undergoing maintenance dialysis in Korea. To our knowledge, this is one of the few studies to comprehensively characterize maternal comorbidities, dialysis practices, obstetric complications, and congenital anomalies in live-born infants in this high-risk population using a nationwide database. Given the rarity of this clinical condition, large-scale prospective studies are inherently difficult to conduct, and our findings may serve as a meaningful foundation for establishing evidence-based clinical guidelines for the management of pregnant women with ESKD.

## Figures and Tables

**Table 1:** Patients’ baseline characteristics.

Characteristics	% (Number/Total Number) or Mean ± STD (Total Number)
Age of child delivery	36.1 ± 4.94 (29)
Dialysis vintage before delivery	
<1 y	27.6 (8/29)
1–2 y	13.8 (4/29)
2–3 y	10.3 (3/29)
3–4 y	13.8 (4/29)
≥4 y	34.5 (10/29)
Vascular Access before delivery in hemodialysis	
Catheter	62.1 (18/29)
AVF	48.3 (14/29)
AVG	0 (0/29)
Medical conditions	
Hypertension	79.3 (23/29)
DM	48.3 (14/29)
Dyslipidemia	89.7 (26/29)
Coronary artery disease	10.3 (3/29)
Heart failure	24.1 (7/29)
Chronic airway disease	6.9 (2/29)
Liver disease	20.7 (6/29)
Systemic lupus erythematosus	6.9 (2/29)
ADPKD	0 (0/29)
HIV/AIDS	0 (0/29)
Kidney transplantation	6.9 (2/29)

Data are expressed as mean ± standard deviation or % (number/total number). AIDS; acquired immunodeficiency syndrome, AVF; arteriovenous fistula, AVG; arteriovenous graft, DM; diabetes mellitus, ADPKD; autosomal dominant polycystic kidney disease, HIV; human immunodeficiency virus.

**Table 2:** Clinical characteristics associated with pregnancy.

	% (Number/Total Number) or Mean ± STD
Multiple gestation	6.9 (2/29)
Type of delivery	
Cesarean section	75.9 (22/29)
Spontaneous delivery	24.1 (7/29)
Timing of conception	
Conception during dialysis	75.9 (22/29)
Conception before starting dialysis	24.1 (7/29)
Number of outpatient clinic visits during pregnancy	121.8 ± 58.8 (*N* = 29)
Number of hospitalizations during pregnancy	2.93 ± 1.86 (*N* = 29)
Type of hospital where the delivery took place	
Primary care	0 (0/29)
Secondary care	6.9 (2/29)
Tertiary care	93.1 (27/29)
Weekly dialysis sessions	
Before pregnancy	2.85 ± 0.34 (*N* = 29)
During pregnancy	3.93 ± 0.91 (*N* = 29)
First trimester	2.88 ± 0.40 (*N* = 29)
Second trimester	3.92 ± 1.94 (*N* = 29)
Third trimester	4.51 ± 1.07 (*N* = 29)
After pregnancy	2.83 ± 0.43 (*N* = 29)
Kidney transplantation after delivery	27.6 (8/29)
Peritoneal Dialysis after delivery	10.3 (3/29)

Data are expressed as mean ± standard deviation or % (number/total number).

**Table 3:** Utilization of healthcare services during the peri-pregnancy period.

% (Number/Total Number)	Preconception Period	Gestation Period	Postpartum Period
Primary care	57.1 (12/21)	44.8 (13/29)	50.0 (13/26)
Secondary care	28.6 (6/21)	20.7 (6/29)	30.8 (8/26)
Tertiary care	14.3 (3/21)	34.5 (10/29)	19.2 (5/26)

Data are expressed as % (number/total number).

**Table 4:** Pregnancy-related maternal complications.

	% (Number/Total Number)
Pregnancy-related complications	
Preeclampsia	17.2 (5/29)
Premature rupture of membranes	13.8 (4/29)
Genitourinary infection during pregnancy	13.8 (4/29)
Abnormal uterine bleeding during pregnancy	10.3 (3/29)
Gestational diabetes	17.2 (5/29)
Delivery-related complications	
False labor	13.8 (4/29)
Fetal presentation abnormality	20.7 (6/29)
Preterm delivery	51.7 (15/29)

Data are expressed as % (number/total number).

**Table 5:** Congenital anomalies in neonates.

	No. of Events (Sample Size)	IR (per 100)
Any malformations	5 (31)	16.13
Nervous system	0 (31)	0
Eye	0 (31)	0
Ear, face, and neck	0 (31)	0
Heart defects (atrial septal defects)	5 (31)	16.13
Respiratory system	0 (31)	0
Oral clefts	0 (31)	0
Digestive system	0 (31)	0
Abdominal wall defects	0 (31)	0
Genitourinary system	0 (31)	0
Limb	0 (31)	0
Other malformations	0 (31)	0

Data are expressed as % (total number). IR, incidence rate per 100 live births.

## Data Availability

Data used in this research are subject to third-party restrictions and are not publicly available. Requests for access may be directed to NHIS in accordance with their data access policies.

## Supplementary Materials

The following supporting information can be downloaded at https://www.mdpi.com/article/10.3390/jcm15124621/s1. Table S1: Codes used to define diagnoses, procedures, and clinical characteristics.
