# Supplementary material for: Pregnancy Outcomes and Associated Complications in Patients Undergoing Hemodialysis and Their Neonates: A Nationwide Study in South Korea (2014–2022)
Source: J Clin Med. 2026 Jun 14;15(12):4621. doi: 10.3390/jcm15124621 (PMC13301050; doi:10.3390/jcm15124621)
Supplement: Supplementary file 1 [file jcm-15-04621-s001.zip › jcm-4330600-supplementary.pdf]

Table S1. Codes used to define diagnoses, procedures, and clinical characteristics

Categories	Codes
**Inclusion criteria**	
Delivery	R3131-R3148, R4351-R4362, R4380, R4507-R4520, R5001-R5002, RA311-RA318, RA361-RA362, and RA380-RA434 (procedure codes)
Hemodialysis	V001 (copayment exemption codes)
**Vascular access**	**Procedure codes**
Catheter	O7011–O7020
AVF	O2081, O2082
AVG	O2084
**Maternal conditions**	**ICD-10 codes (except for kidney transplantation)**
Hypertension	I10–I15 and O16
Diabetes mellitus	E100–E109, E110–E119, E120–E129, E130–E139, and E140–E149, N083, N251, O240–O243, P700, R730, Z131
Dyslipidemia	E785
Coronary artery disease	I200-I214, I240, I251
Heart failure	I110, I119, I130-I132, I500-I502, I509, I971
COPD	J440-J441, J448-J449
Asthma	J450-J451, J458-J459, J46, J828
Liver disease	B160,B162, B169, B171, B178, B180, B182, K702, K703, K709, K717, K746, O984, P788, Q446, Q447
Lupus nephritis	D686, D863, G058, G737, H011,I328, I682, J991, K754, L929-L932, M320-M321, M328, M329, N085, N164
ADPKD	Q612
HIV infection	B200-B213, B217-B222, B227, B230-B232, B238, B24, F024, Z206, Z21
Kidney transplantation	R3280 (procedure code)
**Pregnancy or delivery related complications**	**ICD-10 codes**
Preterm delivery	O601-O603
Preeclampsia	O11
Premature rupture of membranes	O420-O422, O429, O458
Genitourinary infection during pregnancy	O230-O235, O239
Abnormal uterine bleeding	N938-N939
Gestational diabetes	E149, O244, P701
False labor	O470-O471, O479
Fetal presentation abnormality	O321-O323, O326
**Major congenital malformations**	**ICD-10 codes**
Nervous system	Q00-Q07
Eye	Q100, Q104, Q106-Q109, Q11-Q12, Q130-Q134, Q136-Q139, and Q14-Q15
Ear, face, and neck	Q16, Q176-Q178, Q183, and Q188
Heart defects	Defects of cardiac chambers and connections (Q20); cardiac septal defects (Q21); pulmonary and tricuspid valve defects (Q22); aortic and

	mitral valve defects (Q23); other heart defects (Q24); defects of the great arteries (Q25); and defects of the great veins (Q260, Q262-Q269)
Respiratory system	Q300, Q321-Q329, Q330, Q332-Q335, Q337-Q339, and Q34
Oral clefts	Q35-Q37
Digestive system	Tongue, mouth, and pharynx (Q380, Q383-Q389); oesophagus (Q39); upper alimentary tract (Q402-Q409); small intestine (Q41); large intestine (Q42); other malformations of the intestine (Q431-Q439); gallbladder, bile ducts and liver (Q44); other malformations of the digestive system (Q45); and diaphragmatic hernia (Q790)
Abdominal wall defects	Q792-Q793 and Q795
Urinary system	Q60, Q611-Q619, Q620-Q626, Q628-Q629, Q630-Q632, Q634-Q639, Q64, and Q794
Genital organs	Q50-Q51, Q520-Q522, Q524, Q526, Q528-Q529, and Q54-Q56
Limb	Q660-Q661, Q679, Q681-Q682, Q686-Q689, and Q70-Q74
Other malformations	Q750, Q77, Q782-Q788, Q80-Q81, Q820-Q824, Q826-Q829, Q860, Q890, and Q893-Q894

Abbreviations: ICD-10, International Classification of Diseases 10th revision; AVF; arteriovenous fistula, AVG; arteriovenous graft, COPD, Chronic Obstructive Pulmonary Disease; ADPKD, Autosomal Dominant Polycystic Kidney Disease; HIV, Human Immunodeficiency Virus
